# Differentiation and Gene Flow among European Populations of *Leishmania infantum* MON-1

**DOI:** 10.1371/journal.pntd.0000261

**Published:** 2008-07-09

**Authors:** Katrin Kuhls, Carmen Chicharro, Carmen Cañavate, Sofia Cortes, Lenea Campino, Christos Haralambous, Ketty Soteriadou, Francine Pratlong, Jean-Pierre Dedet, Isabel Mauricio, Michael Miles, Matthias Schaar, Sebastian Ochsenreither, Oliver A. Radtke, Gabriele Schönian

**Affiliations:** 1 Institut für Mikrobiologie und Hygiene, Charité Universitätsmedizin Berlin, Berlin, Germany; 2 WHO Collaborating Centre for Leishmaniasis, Servicio de Parasitología, Instituto de Salud Carlos III, Mahadahonda (Madrid), Spain; 3 Unidade de Leishmanioses, Instituto de Higiene e Medicina Tropical, Universidade Nova de Lisboa, Lisboa, Portugal; 4 Laboratory of Molecular Parasitology, Hellenic Pasteur Institute, Athens, Greece; 5 Laboratoire de Parasitologie and Centre National de Référence des Leishmania, Université Montpellier 1 and CHU Montpellier, Montpellier, France; 6 Department of Infectious and Tropical Diseases, London School of Hygiene and Tropical Medicine, London, United Kingdom; Hebrew University, Israel

## Abstract

**Background:**

*Leishmania infantum* is the causative agent of visceral and cutaneous leishmaniasis in the Mediterranean region, South America, and China. MON-1 *L. infantum* is the predominating zymodeme in all endemic regions, both in humans and dogs, the reservoir host. In order to answer important epidemiological questions it is essential to discriminate strains of MON-1.

**Methodology/Principal Findings:**

We have used a set of 14 microsatellite markers to analyse 141 strains of *L. infantum* mainly from Spain, Portugal, and Greece of which 107 strains were typed by MLEE as MON-1. The highly variable microsatellites have the potential to discriminate MON-1 strains from other *L. infantum* zymodemes and even within MON-1 strains. Model- and distance-based analysis detected a considerable amount of structure within European *L. infantum.* Two major monophyletic groups—MON-1 and non-MON-1—could be distinguished, with non-MON-1 being more polymorphic. Strains of MON-98, 77, and 108 were always part of the MON-1 group. Among MON-1, three geographically determined and genetically differentiated populations could be identified: (1) Greece; (2) Spain islands–Majorca/Ibiza; (3) mainland Portugal/Spain. All four populations showed a predominantly clonal structure; however, there are indications of occasional recombination events and gene flow even between MON-1 and non-MON-1. Sand fly vectors seem to play an important role in sustaining genetic diversity. No correlation was observed between *Leishmania* genotypes, host specificity, and clinical manifestation. In the case of relapse/re-infection, only re-infections by a strain with a different MLMT profile can be unequivocally identified, since not all strains have individual MLMT profiles.

**Conclusion:**

In the present study for the first time several key epidemiological questions could be addressed for the MON-1 zymodeme, because of the high discriminatory power of microsatellite markers, thus creating a basis for further epidemiological investigations.

## Author Summary

Visceral leishmaniasis is caused by protozoan parasites of the genus *Leishmania*. This disease is a public health problem in countries bordering the Mediterranean, in China, and South America. Until now, isoenzyme analysis, a method with several advantages but also some limitations, is the gold standard for typing the causative agent *L. infantum*. We have developed a new method based on hypervariable DNA markers, the microsatellites. Its higher discriminatory power, genotype-based analysis, the possibility to use biological material instead of parasite cultures, and the fast analysis are the major improvements. We could demonstrate for the first time that there exist different geographically determined populations within the predominant zymodeme of *L. infantum*, which has important epidemiological implications. We also tested for relationships between genotype and clinical picture and/or host background. *Leishmania* is considered to reproduce mainly clonally; however, we found some indication for recombination in our study. Our work constitutes a solid basis for further population and epidemiological studies of *L. infantum* by completing the existing microsatellite database by analysing strains from other endemic foci.

## Introduction

Visceral leishmaniasis (VL) caused by *Leishmania infantum* (synonym *L. chagasi,* [1]) is a public-health problem in most countries bordering the Mediterranean, China and South America. Currently, the epidemiology of Mediterranean VL is changing. Increasing incidence [2] and a shift in the bulk of cases from children to adults [3],[4], related to the emergence of HIV, has been reported. Since 1985 up to 80% of the cases have occurred in immunocompromised adults [5],[6]. *Leishmania*/HIV co-infections have become increasingly frequent in Southern Europe, particularly in Spain, France, and Italy, with 25–70% of adult cases being related to HIV infection and up to 9% of AIDS cases developing VL [7],[8]. In addition to the classical vector-based disease transmission, an anthroponotic cycle has emerged among intravenous drug users where syringes replace the sand fly vector [9]–[14]. Recently, *L. infantum* parasites have been found to have spread northward in continental Italy perhaps due to climatic changes [15]–[17]. Dogs are the main reservoir hosts for *L. infantum,* being part of the domestic (pet dogs) and peridomestic (stray dogs and wild canids) transmission cycles. The prevalence of canine leishmaniasis is high in all European Mediterranean countries [18]–[20].

The gold standard method for typing *Leishmania* is still Multilocus Enzyme Electrophoresis (MLEE, isoenzyme analysis). Most widely used is the Montpellier system (MON) which is based on the analysis of 15 enzymes [21]. *Leishmania infantum* is characterized by a broad enzymatic polymorphism. At present this species includes 31 zymodemes of which 30 have been found in humans [22]. Some of them were related to VL only (e.g. MON-27, 28, 72, 77, 187), others only to cutaneous leishmaniasis (CL) (e.g. MON-11, 29, 33, 78, 111), and few were isolated from both VL and CL cases (e.g. MON-1, 24, 34, 80). In *L. infantum*/HIV co-infections the tropism of some zymodemes (MON-24, 29, 33, 78) has changed from CL to VL [23]–[25].

MON-1 is the most prevalent zymodeme; it occurs in more than 30 countries worldwide, and represents approximately 70% of all identified strains [26]. In Europe, it varies between 88% in Southern France [25], over 50% in Italy [27], 96.7% in Portugal [28] and 44–58% in Spain [9],[29],[30]. Up to 73% of HIV/VL co-infections in Europe are due to this zymodeme [24]. In immunocompetent patients MON-1 causes up to 90% of VL cases, but only 20% of CL cases [11]. MON-1 is also the prevalent zymodeme in dogs [19],[25],[29] whilst other zymodemes such as MON-98, 108, 253, 77, and 24 are found occasionally. In contrast, MON-1 has only been detected in 18% of the sand fly samples [29].

Epidemiological studies on VL caused by *L. infantum* require the use of techniques that are able to differentiate MON-1 strains. The first indications for heterogeneity among MON-1 strains were based on RAPD analyses [31]–[34], analysis of three microsatellite markers [35], PCR-RFLP of the intergenic *cpb* and intragenic *gp63* regions [36] and RFLP analysis of minicircle kDNA [37],[38].

Microsatellites are tandemly repeated stretches of short nucleotide motives of 1–6 bp ubiquitously distributed in eukaryotic genomes. They mutate at rates five to six orders of magnitude higher than the bulk of DNA. These highly polymorphic and co-dominant markers have been shown to be very useful for population studies [39] and have been applied for a number of species, among them quite recently *L. tropica* [40] and the *L. donovani* complex [41]. A comparison of different genotyping methods targeting *Leishmania* DNA regions with different molecular clocks [42] revealed that kDNA PCR-RFLP and multilocus microsatellite typing (MLMT) were the most powerful tools for MON-1 strain tracking.

In the present study we performed MLMT using a set of 14 microsatellite markers for 141 strains of *L. infantum* of different zymodemes with strong sampling emphasis on MON-1, mainly from Spain, Portugal and Greece in order to investigate the population structure and dynamics in the corresponding natural foci. We also attempted to correlate microsatellite patterns with host specificity and manifestation of the disease. Strains from recurrent infections were included to test whether MLMT was able to differentiate between relapse and re-infection.

## Materials and Methods

### Parasite cultures and DNA extraction

Sources, designation, geographical origins, MLEE identification and clinical manifestation of the *Leishmania infantum* strains are listed in **Table 1**. The 66 Spanish strains were collected from humans and dogs in four regions-Madrid, Ibiza, Majorca, and Catalonia. Forty four strains, from humans, dogs and sand fly vectors, were obtained from four Portuguese regions: Metropolitan region of Lisbon, Alentejo, Algarve, Alto Douro. The 16 human and canine strains from Greece are originating from two foci-Athens and Crete. The seven strains from France were collected in four of the five known endemic foci: Cévennes, Côte d'Azur, Provence, and Pyrénées-Orientales. Additional strains or the respective DNA samples were obtained from the following culture collections: Centre National de Référence des *Leishmania*, Montpellier, France; KIT (Royal Tropical Institute), Amsterdam, Netherlands; WHO's Jerusalem Reference Centre for Leishmaniases, Hebrew University–Hadassah Medical School, Jerusalem, Israel; London School of Hygiene and Tropical Medicine, London, UK. The strain set included isolates from 12 relapse cases. Ten of them represented two episodes of infection (original infection and relapse), one three and another one four episodes. All parasites were cultivated as described previously [37],[43],[44]. DNA was isolated using proteinase K-phenol/chloroform extraction [45], suspended in TE-buffer or distilled water and stored at 4°C until use.

**Table 1:** Designation and characteristics of *Leishmania infantum* strains used in this study

Country (number of strains)	Region (number of MON-1 strains of each region)	MON	Pathology^a^	Lab code	WHO-Code	Population assignment *K* = 4
Spain	Majorca (12)	1	VL/HIV+	INF-41	MHOM/ES/1993/PM1	2
(66)	Majorca	1	VL/HIV+	ES11(I)	MHOM/ES/2001/LLM-981	2
	Majorca	1	2. Episode of ES11(I)	ES12(I)	MHOM/ES/2002/LLM-1122	2
	Majorca	1	VL/HIV+	ES13(I)	MHOM/ES/2001/LLM-1048	3
	Majorca	1	VL/HIV+	ES14(I)	MHOM/ES/2001/LLM-1049	3
	Majorca	1	CL/HIV+	ES15(I)	MHOM/ES/2002/LLM-1150	2
	Majorca	1	VL	ES16(I)	MHOM/ES/2002/LLM-1109	3
	Majorca	1	CanL	ES17(I)	MCAN/ES/2001/LLM-1008	2
	Majorca	1	CanL	ES18(I)	MCAN/ES/2001/LLM-1007	2
	Majorca	1	CanL	ES19(I)	MCAN/ES/2001/LLM-1038	2
	Majorca	1	3. Episode of ES11(I)	ES13(III)	MHOM/ES/2001/LLM-1035	2
	Majorca	1	4. Episode of ES11(I)	ES14(III)	MHOM/ES/2002/LLM-1167	2
	Ibiza (15)	1	CanL	ES20(I)	MCAN/ES/2002/LLM-1149	2
	Ibiza	1	CanL	ES21(I)	MCAN/ES/2002/LLM-1155	2
	Ibiza	1	CanL	ES22(I)	MCAN/ES/2002/LLM-1203	2
	Ibiza	1	CanL	ES23(I)	MCAN/ES/2002/LLM-1139	2
	Ibiza	1	CanL	ES24(I)	MCAN/ES/2002/LLM-1141	2
	Ibiza	1	CanL	ES25(I)	MCAN/ES/2002/LLM-1158	2
	Ibiza	1	CanL	ES10(II)	MCAN/ES/2003/LLM-1228	2
	Ibiza	1	CanL	ES11(II)	MCAN/ES/2003/LLM-1233	2
	Ibiza	1	CanL	ES12(II)	MCAN/ES/2003/LLM-1238	2
	Ibiza	1	CanL	ES13(II)	MCAN/ES/2003/LLM-1240	2
	Ibiza	1	CanL	ES14(II)	MCAN/ES/2001/LLM-1215	2
	Ibiza	1	CanL	ES15(II)	MCAN/ES/2003/LLM-1237	2
	Ibiza	1	CanL	ES16(II)	MCAN/ES/2003/LLM-1241	2
	Ibiza	1	CanL	ES17(II)	MCAN/ES/2003/LLM-1226	2
	Ibiza	1	CanL	ES18(II)	MCAN/ES/2003/LLM-1267	2
	Madrid (20)	1	VL/transplant.	ES1(I)	MHOM/ES/2001/LLM-984	4
	Madrid	1	VL/HIV+	ES2(I)	MHOM/ES/2001/LLM-983	3
	Madrid	1	VL	ES3(I)	MHOM/ES/2001/LLM-980	4
	Madrid	1	VL/HIV+	ES4(I)	MHOM/ES/2002/LLM-1181	3
	Madrid	1	2. Episode of ES4(I)	ES5(I)	MHOM/ES/2002/LLM-1212	3
	Madrid	1	VL/HIV+	ES6(I)	MHOM/ES/2002/LLM-1166	4
	Madrid	1	CanL	ES7(I)	MCAN/ES/2001/LLM-1006	3
	Madrid	1	CanL	ES8(I)	MCAN/ES/2001/LLM-1014	3
	Madrid	1	CanL	ES9(I)	MCAN/ES/2001/LLM-1037	2
	Madrid	1	CanL	ES10(I)	MCAN/ES/2001/LLM-1040	3
	Madrid	1	VL/HIV+	ES1(III)	MHOM/ES/2002/LLM-1220	2
	Madrid	1	2. Episode of ES1(III)	ES2(III)	MHOM/ES/2002/LLM-1217	2
	Madrid	1	CanL	ES1(II)	MCAN/ES/2001/LLM-1068	3
	Madrid	1	CanL	ES2(II)	MCAN/ES/2001/LLM-1102	1
	Madrid	1	CanL	ES3(II)	MCAN/ES/2001/LLM-1106	3
	Madrid	1	CanL	ES5(II)	MCAN/ES/2002/LLM-1113	3
	Madrid	1	CanL	ES6(II)	MCAN/ES/2001/LLM-1116	3
	Madrid	1	CanL	ES7(II)	MCAN/ES/2001/LLM-1128	3
	Madrid	1	CanL	ES8(II)	MCAN/ES/2001/LLM-1136	3
	Madrid	1	CanL	ES9(II)	MCAN/ES/2001/LLM-1148	2
	Catalonia (3)	1	CL	INF-43	MHOM/ES/1986/BCN16	3
	Catalonia	1	VL	ES7(III)	MHOM/ES/2002/LLM-1184	3
	Catalonia	1	2. Episode of ES7(III)	ES8(III)	MHOM/ES/2003/LLM-1232	3
	Madrid	198	VL/HIV+	INF-37	MHOM/ES/88/LLM175	4
	Madrid	199	VL/HIV+	INF-55	MHOM/ES/92/LLM373	4
	Catalonia	77	CanL	INF-32	MCAN/ES/86/LEM935	3/4
	Valencia	183	VL/HIV+	INF-46	MHOM/ES/91/LEM2298	4
	Madrid	24	VL/HIV+	ES5(III)	MHOM/ES/97/LLM-707	4
	Madrid	24	2. Episode of ES5(III)	ES6(III)	MHOM/ES/97/LLM-709	4
	Majorca	24	VL/HIV+	ES9(III)	MHOM/ES/98/LLM-810	2/4
	Majorca	24	2. Episode of ES9(III)	ES10(III)	MHOM/ES/99/LLM-846	2/4
	unknown	24	CL	INF-04	MHOM/ES/87/Lombardi	4
	Madrid	27	VL/HIV+	ES3(III)	MHOM/ES/2001/LLM-1036	4
	Madrid	27	2. Episode of ES3(III)	ES4(III)	MHOM/ES/2003/LLM-1254	4
	Andalucia	34	VL/HIV+	ES11(III)	MHOM/ES/98/LLM-745	4
	Andalucia	34	2. Episode of ES11(III)	ES12(III)	MHOM/ES/99/LLM-879	4
	Madrid	34	VL/HIV+	ES15(III)	MHOM/ES/95/LLM-531	4
	Madrid	34	2. Episode of ES15(III)	ES16(III)	MHOM/ES/98/LLM-780	4
	Madrid	34	3. Episode of ES15(III)	ES17(III)	MHOM/ES/2001/LLM-1034	4
Portugal	Alto Douro (10)	1	CanL	PT5(I)	MCAN/PT/1989/IMT162	3
(44)	Alto Douro	1	*Phlebotomus*	PT6(I)	IARI/PT/1989/IMT169	2
	Alto Douro	1	*Phlebotomus*	PT7(I)	IARI/PT/1989/IMT170	2
	Alto Douro	1	VL	PT8(I)	MHOM/PT/2002/IMT279	3
	Alto Douro	1	VL	PT9(I)	MHOM/PT/2002/IMT288	3
	Alto Douro	1	CL	PT10(I)	MHOM/PT/2003/IMT337	3
	Alto Douro	1	VL	PT7(II)	MHOM/PT/2004/IMT359	3
	Alto Douro	1	VL	PT8(II)	MHOM/PT/2004/IMT360	3
	Alto Douro	1	CanL	PT13(II)	MCAN/PT/1989/IMT160	2
	Alto Douro	1	CanL	PT14(II)	MCAN/PT/1989/IMT161	3
	Algarve (3)	1	CanL	PT1(I)	MCAN/PT/1993/IMT193	3
	Algarve	1	*Phlebotomus*	PT2(I)	IPERN/PT/1993/IMT189	3/4
	Algarve	1	CanL	PT17(II)	MCAN/PT/1994/IMT204	3
	Alentejo (4 MON-1)	1	CanL	PT3(I)	MCAN/PT/1995/IMT205	3
	Alentejo	1	CanL	PT4(I)	MCAN/PT/2003/IMT328	3
	Alentejo	n.d.	VL	PT9(II)	MHOM/PT/2004/IMT363	4
	Alentejo	1	CanL	PT15(II)	MCAN/PT/2004/IMT355	3
	Alentejo	1	CanL	PT16(II)	MCAN/PT/2004/IMT356	3
	Lisbon-MRL (25)^b^	1	CL	INF-44	MHOM/PT/2000/IMT260	3
	Lisbon-MRL	1	CanL	PT11(I)	MCAN/PT/1997/IMT229	3
	Lisbon-MRL	1	CanL	PT12(I)	MCAN/PT/2003/IMT300	3
	Lisbon-MRL	1	CanL	PT13(I)	MCAN/PT/2003/IMT327	3
	Lisbon-MRL	1	CanL	PT14(I)	MCAN/PT/2003/IMT316	3
	Lisbon-MRL	1	CanL	PT15(I)	MCAN/PT/2003/IMT329	3
	Lisbon-MRL	1	CanL	PT16(I)	MCAN/PT/2003/IMT330	3
	Lisbon-MRL	1	CanL	PT17(I)	MCAN/PT/2003/IMT338	3
	Lisbon-MRL	1	CanL	PT18(I)	MCAN/PT/2003/IMT339	3
	Lisbon-MRL	1	VL/HIV+	PT19(I)	MHOM/PT/1989/IMT163	3
	Lisbon-MRL	1	VL/HIV+	PT20(I)	MHOM/PT/2002/IMT293	3
	Lisbon-MRL	1	2. Episode of PT20(I)	PT21(I)	MHOM/PT/2003/IMT293-B	3
	Lisbon-MRL	1	VCL/HIV+	PT22(I)	MHOM/PT/2002/IMT294	3
	Lisbon-MRL	1	VL/HIV+	PT23(I)	MHOM/PT/2002/IMT296	3
	Lisbon-MRL	1	VL/HIV+	PT24(I)	MHOM/PT/2003/IMT299	3
	Lisbon-MRL	1	VL/HIV+	PT1(II)	MHOM/PT/2000/IMT262	3
	Lisbon-MRL	1	2. Episode of PT1(II)	PT2(II)	MHOM/PT/2000/IMT262-A	3
	Lisbon-MRL	1	VL/HIV+	PT3(II)	MHOM/PT/1993/IMT184	3
	Lisbon-MRL	1	VL	PT4(II)	MHOM/PT/1988/IMT151	3
	Lisbon-MRL	1	VCL/HIV+	PT5(II)	MHOM/PT/2004/IMT362	3
	Lisbon-MRL	1	VL/HIV+	PT6(II)	MHOM/PT/2004/IMT364	3
	Lisbon-MRL	1	CanL	PT10(II)	MCAN/PT/2003/IMT329	3
	Lisbon-MRL	1	CanL	PT11(II)	MCAN/PT/2003/IMT331	3
	Lisbon-MRL	1	CanL	PT12(II)	MCAN/PT/2003/IMT354	3
	Lisbon-MRL	1	fox	PT18(II)	VUL/PT/1982/IMT108	3
	Lisbon-MRL	80	VL/HIV+	PT25(I)	MHOM/PT/98/IMT238	4
France	Cévennes (2)	1	VL	INF-39	MHOM/FR/78/LEM75	3
(7)	Cévennes	1	CL	INF-42	MHOM/FR/97/LSL29	3
	Côte d'Azur (1)	1	VL	INF-40	MHOM/FR/95/LPN114	1
	Provence	108	CanL	INF-35	MCAN/FR/87/RM1	2
	Pyrénées-Orientales	29	CL	INF-45	MHOM/FR/96/LEM3249	4
	Pyrénées-Orientales	11	CL	INF-47	MHOM/FR/80/LEM189	4
	unknown	n.d.	VL	INF-03	MHOM/FR/62/LRC-L47	4
						
Italy	unknown	228	VL/HIV+	INF-56	MHOM/IT/94/ISS1036	4
(2)	Sicily	188	VL/HIV+	INF-57	MHOM/IT/93/ISS800	4
Malta	Malta	78	CL	INF-48	MHOM/MT/85/BUCK	4
(1)						
Greece	Crete (4)	1	VL	GR3	MHOM/GR/2001/GH3	1
(16)	Crete	1	VL	GR4	MHOM/GR/2001/GH5	1
	Crete	1	CanL	GR14	MCAN/GR/2001/GD7	1
	Crete	1	VL	GR16	MHOM/GR/2002/GH12	1
	Crete	98	CanL	GR13	MCAN/GR/2003/GD5	1
	Crete	98	CanL	GR11	MCAN/GR/2001/GD3	1
	Crete	98	CanL	GR12	MCAN/GR/2001/GD4	1
	Crete	98	CanL	GR15	MCAN/GR/2001/GD8	1
	Athens (7)	1	VL	GR1	MHOM/GR/2001/GH1	1
	Athens	1	VL	GR2	MHOM/GR/2001/GH2	1
	Athens	1	VL 2. Episode of GR5	GR7	MHOM/GR/2001/GH8	1
	Athens	1	VL	GR8	MHOM/GR/2001/GH9	1
	Athens	1	VCL/HIV+	GR9	MHOM/GR/2001/GH10	1
	Athens	1	VL	GR10	MHOM/GR/2001/GH11	1
	Athens	1	VL	GR17	MHOM/GR/1978/L4	3
	Athens	98	VL	GR5	MHOM/GR/2001/GH6	1
Turkey	unknown	n.d.	CanL	INF-10	MCAN/TR/96/EP16	1
(2)	unknown	n.d.	unknown	INF-11	MHOM/TR/94/EP3	1
Israel	unknown	n.d.	CanL	INF-12	MCAN/IL/94/LRC-L639	1
(2)	unknown	n.d.	CanL	INF-13	MCAN/IL/96/LRC-L685	1
Tunisia	unknown	1	VL	INF-01^R^	MHOM/TN/80/IPT1^R^	1
(1)						

a VL-Visceral leishmaniasis, CL-Cutaneous leishmaniasis, VCL-Viscero-cutaneous leishmaniasis, CanL-Canine leishmaniasis;

b Metropolitan Region of Lisbon;

R WHO-reference strain; n.d. not defined.

### PCR amplification assays and electrophoretic analysis of the microsatellite markers

A set of fourteen primer pairs were used for microsatellite amplification (**Table 2**) as previously described [41],[46]. Fragments containing single microsatellites were analyzed by either MetaPhor agarose gel electrophoresis, PAGE or capillary electrophoresis. Four percent MetaPhor agarose gels (BioWhittaker Molecular Applications, USA) were used basically for a pre-screening of the strains for polymorphisms [46]. For PAGE 6 to 15 μl of the PCR product were mixed with loading buffer and run under non-denaturating conditions on 12% polyacrylamide gels at 1 kV for 6 h. Gels were silver stained and dried [47]. For high throughput studies PCR products from amplified microsatellites were analyzed with the fragment analysis tool of the CEQ 8000 automated genetic analysis system of Beckman Coulter, USA [46], using fluorescence-conjugated forward primers (Proligo, France) for microsatellite amplification.

**Table 2:** Characteristics of the 14 microsatellite markers used for population analysis of Mediterranean *Leishmania infantum*

Nr.	Marker	Population	n	Repeat array	Fragment size array [bp]	A	*H*_e_	*H*_o_	*F*_IS_
1	Lm2TG	MON-1	113	TG12–27	116–146	9	0.758	0.071	0.906
		non-MON-1	26	TG 12–29	116–150	11	0.848	0.154	0.821
2	TubCA	MON-1	113	CA9–13	80–88	2	0.018	0	1.000
		non-MON-1	26	CA 9–16	80–94	5	0.523	0.038	0.928
3	Lm4TA	MON-1	113	TA 4–16	63–87	9	0.768	0.036	0.954
		non-MON-1	26	TA 9–14	73–83	4	0.533	0.038	0.929
4	Li 41–56	MON-1	113	CA 9–12	88–94	3	0.155	0.009	0.943
		non-MON-1	26	CA 9–13	88–96	4	0.338	0.077	0.776
5	Li 46–67	MON-1	113	CA 6–9	74–80	2	0.018	0.018	−0.004
		non-MON-1	26	CA 6–9	74–80	4	0.709	0.231	0.679
6	Li 22–35	MON-1	113	CA 10–25	88–118	10	0.690	0.080	0.885
		non-MON-1	26	CA 6–28	80–124	13	0.876	0.192	0.784
7	Li 23–41	MON-1	113	GT 7–17	67–87	5	0.294	0.027	0.910
		non-MON-1	26	GT 15–23	83–99	8	0.786	0.269	0.662
8	Li 45–24	MON-1	113	CA 13–22	101–119	6	0.505	0.044	0.913
		non-MON-1	26	CA 7–20	89–115	6	0.643	0.077	0.882
9	Li 71–33	MON-1	113	TG 11–12	105–107	2	0.052	0	1.000
		non-MON-1	26	TG 11–27	105–137	7	0.534	0.231	0.573
10	Li 71-5/2	MON-1	99	CA 8–16	108–124	4	0.302	0.020	0.933
		non-MON-1	26	CA 7–9	106–110	3	0.274	0	1.000
11	Li 71-7	MON-1	113	CA 12–14	98–102	3	0.148	0.035	0.761
		non-MON-1	26	CA 8–13	90–100	5	0.518	0.038	0.927
12	CS20	MON-1	113	TG 17–19	81–85	3	0.331	0	1.000
		non-MON-1	26	TG 18–22	83–91	5	0.750	0.154	0.798
13	LIST7031	MON-1	113	CA 10–12	109–113	3	0.353	0.045	0.874
		non-MON-1	26	CA 10–12	109–113	3	0.528	0.077	0.857
14	LIST7039	MON-1	113	CA 14–17	205–211	3	0.165	0.018	0.892
		non-MON-1	26	CA 15–20	207–217	6	0.729	0.077	0.896
overall		MON-1	112			4.6	0.325	0.029	0.912
		non-MON-1	26			5.6	0.614	0.118	0.810

A, number of alleles; n, sample size; *H*_o_, observed heterozygosity; *H*_e_, expected heterozygosity; *F*_IS_, inbreeding coefficient. Two of the four heterozygous strains with mixed MON-1/non-MON-1 alleles (PT2(I) and INF-32) have been removed from the data set, in case of ES9(III) and ES10(III) only the non-MON-1 genotype has been considered, as these strains have been identified as MON-24 by MLEE.

### Data analysis

Population structure was investigated by the STRUCTURE software [48], which applies a Bayesian model-based clustering approach. This algorithm identifies genetically distinct populations on the basis of allele frequencies. Genetic clusters are constructed from the genotypes identified, estimating for each strain the fraction of its genotype that belongs to each cluster. This clustering method proved superior to distance-based approaches for processing data sets of low variability like those presented by *L. infantum* MON-1. The following parameters were used: burning period of 20,000 iterations, probability estimates based on 200,000 Markov Chain Monte Carlo iterations. The most appropriate number of populations was determined by comparing log-likelihoods for values of *K* between 1 and 16. The log-likelihood values were compared in a diagram. At the plateau (maximum) of the derived Gaussian graph the value of *K* captures the major structure of the populations. In addition we calculated Δ*K*, which is based on the rate of change in the log probability of data between successive *K* values [49]. Ancestral source populations were identified by decreasing the number of *K*.

Phylogenetic analysis was based on microsatellite genetic distances, calculated with the program MICROSAT [50] for the numbers of repeats within each locus using two different measures: D_AS_ (D_ps_), based on the proportion of shared alleles [51] and Chord-distance [52]. Both distances follow the infinite allele model (IAM). Neighbor-joining trees (NJ) of both distance matrices were constructed in PAUP, version 4.0b8 [53]. Confidence intervals were calculated by bootstrapping (100 replications) [54] using the program POPULATIONS 1.2.28 (http://www.legs.cnrs-gif.fr/bioinfo/populations).

For visualising the genetic substructure at population and individual level we applied a factorial correspondence analysis (FCA) implemented in the GENETIX software [55]. This test places the individuals according to the similarity of their allelic state in a three dimensional space. Microsatellite markers as well as populations were analyzed with respect to diversity of alleles (A), expected (gene diversity) and observed heterozygosity (*H*_e_ and *H*_o_, respectively), and the inbreeding coefficient *F*_IS_ applying GDA (http://hydrodictyon.eeb.uconn.edu/people/plewis/software.php) and GENEPOP 3.4 [56].

Genetic differentiation and gene flow [57] was assessed by F-statistics calculating the *F*_ST_ (theta) values (IAM) [58] with the corresponding p-values (confidence test) using the MSA software [59]. Migration rates (gene flow) Nm were calculated as *Nm* = 0.25 (1-*F*_ST_)/*F*_ST_ [60]. Indices of association (I_A_) were calculated to test each population for clonality and recombination using MULTILOCUS [61].

### Ethical considerations

#### Strains collected in Greece

The strains were isolated from the human patients' blood during the process of laboratory diagnosis of the disease at the University Hospital in Heraklion, Crete. The patients were aware that their blood samples were needed for diagnosis of the disease using serology and/or molecular diagnostic methods, isolation of the parasite being the gold standard. Doctors obtained the written consent of the patients. Both the study and the protocols used were approved by the Ethical Scientific Committee of the Medical School of the University of Crete.

#### Strains collected in Portugal

The Portuguese strains are from the IHMT cryobank and were obtained by the diagnosis service that the Leishmaniosis Unit has with the hospitals. All studies involving these strains were approved be the ethical committee of the IHMT. They were included in a previous publication [28], which included the ethical approval. The present study was approved by the Ethics Committee of the Instituto de Higiene e Medicina Tropical, Universidade Nova de Lisboa.

#### Strains collected in Spain

Most of the strains from human were isolated from patients included in two different clinical trials, both previously approved by the corresponding Ethical Committees of the Hospitals taking part in the trials (Hospital Son Dureta (Palma de Mallorca), Fundación Jiménez Díaz (Madrid), Hospital Universitario Vall d'Hebrón (Barcelona) and Hospital Universitario Virgen del Rocío (Sevilla)). All strains isolated from biological samples were received by the laboratory (the Reference Laboratory for Leishmaniasis for the hospitals of the National Health System) in order to carry out a diagnostic identification requested by the physicians. For this diagnostic activity an informed consent and ethical clearance is not needed in Spain. In the case of canine leishmaniasis, the biological samples were also obtained with diagnostic purposes.

#### Strains received from the strain collection in Montpellier, France

In the sampling of strains referring to this paper, the human strains from Montpellier are old strains isolated between 1962 and 1996, at least more than 10 years ago, which have already been object of many publications. Some of those strains are reference strains of zymodemes, as in the case of the *L. infantum* WHO reference strain IPT1. The human strains received in the Montpellier laboratory from other French laboratories, are received for identification purpose, within a diagnostic approach of a physician. This is why the required consent of the patient is a tacit agreement, as usual for biological diagnosis, and not a written consent (written consent in France is required only for a few diagnostics). The identification is carried out for medical purpose and lead consequently to isolation of the parasite. This parasite (the strain) is a foreign element obtained from the patient's body, on which the patient has no rights. The rights attached to human body do not include foreign elements extracted from it. This is the reason for what there is no need of patient consent for using the strains. These arguments were provided in 2005 by the juristic department of INSERM (France).

#### Strains received from the strain collection in London (LSTMH), UK

Similarly as in the case of the strains from France the human strains from London are old strains isolated many years ago, and stored in the cryobank. They also have already been object of many publications.

## Results

### Population structure of European *L. infantum*: genetic diversity, differentiation and gene flow

**Figure 1:**
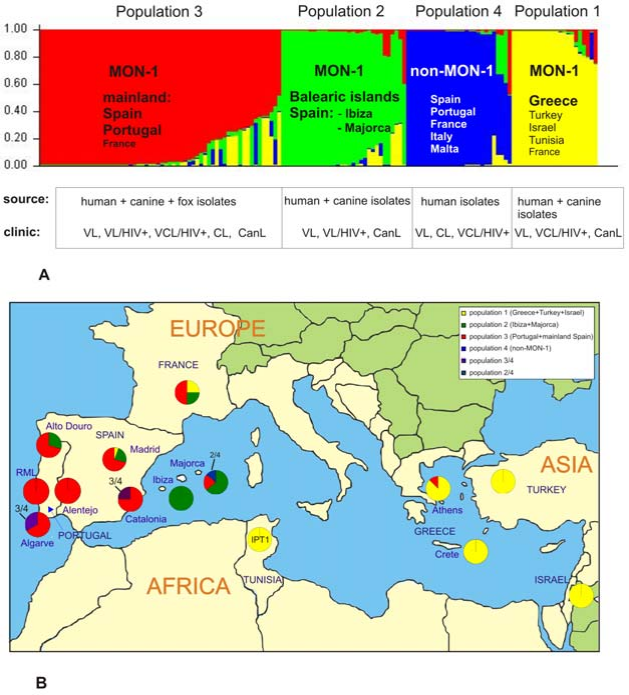
Population structure of Mediterranean *L. infantum* as inferred by STRUCTURE on the basis of data for 14 microsatellite markers obtained for 141 strains. (A) Barplot for *K* = 4-each of the strains is represented by a single vertical line divided into *K* colors, where *K* is the number of populations assumed. Each color represents one population, and the length of the colors segment shows the strain's estimated proportion of membership in that population. (B) Distribution of Mediterranean *L. infantum* MON-1 strains belonging to populations 1–3 in the respective endemic foci. Pie-charts show the proportion of each population sampled in the respective geographical region. Colors correspond to the population specific ones in Figure 1A.

**Figure 2:**
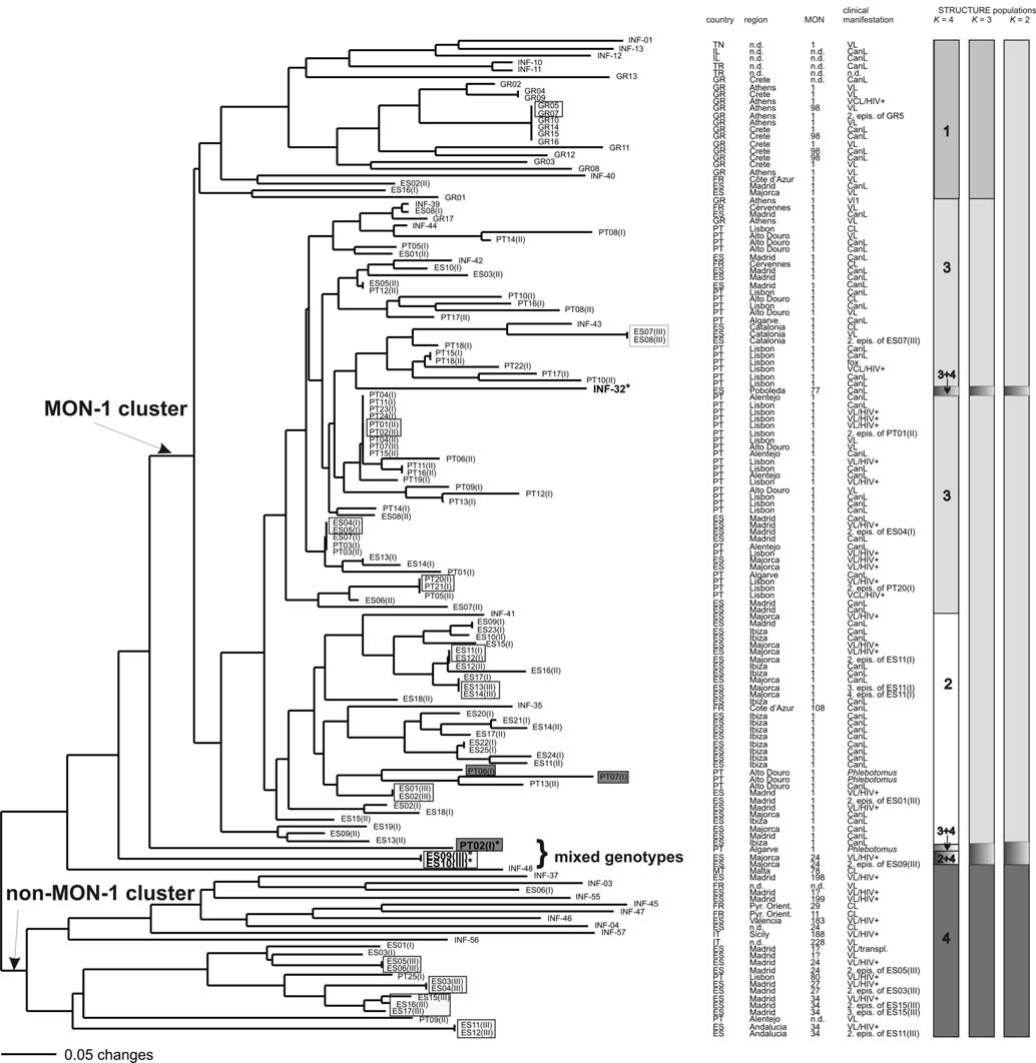
Neighbor-joining tree inferred from Dps-distances calculated for the data of 14 microsatellite markers and all 141 *L. infantum* strains. Geographical origins, zymodemes and clinical manifestation are shown. *Phlebotomus* strains are marked by grey rectangles, relapses by white rectangles. Four strains with mixed MON-1/non-MON-1 ancestry are marked by an asterix. Populations as inferred by STRUCTURE *K* = 2, *K* = 3 and *K* = 4 are indicated by bars in different colors next to the tree. Midpoint rooting was applied for the NJ tree, no outgroup has been used.

A total of 106 different MLMT genotypes based on 14 microsatellite markers were identified for 141 strains of *L. infantum* mostly from Southern Europe. A Bayesian model-based clustering algorithm implemented in the software STRUCTURE was used to infer the population structure of European *L. infantum* based on these MLMT data. According to Δ*K,* the most probable number of populations for the complete data set of 141 strains was four: (1) MON-1 from Greece, Turkey, Israel, and Tunisia (+1 strain from France); (2) MON-1 from Majorca and Ibiza (Spain); (3) MON-1 from Portugal and mainland Spain; and (4) non-MON-1 strains from Spain, Portugal, Italy, Malta **(** **Figure 1** **)**. Ancestral source populations were identified by successively increasing the number of populations (*K*) from 2 as indicated by the bars next to the tree in **Figure 2**. The first and most important split at *K* = 2 divided non-MON-1 from MON-1 strains. The MON-1 cluster was subdivided at *K* = 3 into a group of Iberian strains, which included strains from France, and a group comprising strains from Greece, Turkey, Israel, and Tunisia. Finally, at *K* = 4, the Iberian group split into mainland and Balearic Islands (**Table 1**).

One hundred and fifteen strains representing 84 distinct genotypes formed the MON-1 group and 26 strains representing 22 genotypes the non-MON-1 group, respectively. The assignment of strains to these groups was not always compatible with zymodeme identification: three Spanish MON-1 strains ES1(I), ES3(I), ES6(I), clustered with non-MON-1 strains, whereas strains INF-32 (MON-77), INF-35 (MON-108) and all Greek MON-98 strains grouped with the MON-1 strains. Populations as defined by STRUCTURE were used for all subsequent population genetics analyses.

In addition to the model-based algorithm, we used a genetic distance-based approach to infer the population structure of European *L. infantum* (**Figure 2**). The same 4 major populations as defined by STRUCTURE were detected, as indicated by bars, however they were not supported by significant bootstrap values (data not shown). This may be due to the extremely high similarity of these strains, a certain amount of homoplasy and the existence of strains with mixed or intermediate genotypes. Bootstrap values >50% were obtained only for nodes between some subgroups inside the major clusters. The identical results obtained by two different methods, however, strongly support the existence of these particular main populations. The MON-1 and non-MON-1 clusters were monophyletic. Zymodemes MON-77, 108 and 98 were, again, members of the MON-1 cluster. There were two subclusters of non-MON-1 strains that were also observed in STRUCTURE at *K* = 5 (data not shown): one comprises predominantly MON-34, MON-27 and MON-80, the other MON-11, 29, 183, 188, 198, 199, 228. MON-24 is present in both subclusters. Long branches in the non-MON-1 group indicated a higher diversity among these strains.

The number of microsatellite alleles ranged from 2–10 (mean 4.6) for the MON-1 strains, and from 3–13 (mean 5.6) for the non-MON-1 strains (**Table 2**). The most variable markers were Li 22–35 and Lm2TG, the least variable ones Li 46–67 and TubCA. The observed heterozygosity (*H*_o_) varied between 0–0.08 for MON-1 strains, and 0–0.269 for non-MON-1 strains, indicating that most microsatellite loci were heterozygous in at least one strain, with a higher degree of heterozygosity in non-MON-1 strains. The expected heterozygosity (*H*_e_) as a measure of genetic diversity was between 0.018–0.768 (MON-1) and 0.274–0.876 (non-MON-1), and, in most of the markers, much higher than the mean *H*_o_ (0.029 MON-1 and 0.118 non-MON-1). The mean of the inbreeding coefficients was 0.912 and 0.81 for MON-1 and non-MON-1 strains, respectively, pointing to a homozygotes predominance. The non-MON-1 strains were more diverse, with greater distances on the Neighbor-joining tree, higher numbers of allelic variants and higher values of diversity measures, in spite of the much smaller number of strains, perhaps reflecting the combination of distinct zymodemes in this group.

**Figure 3:**
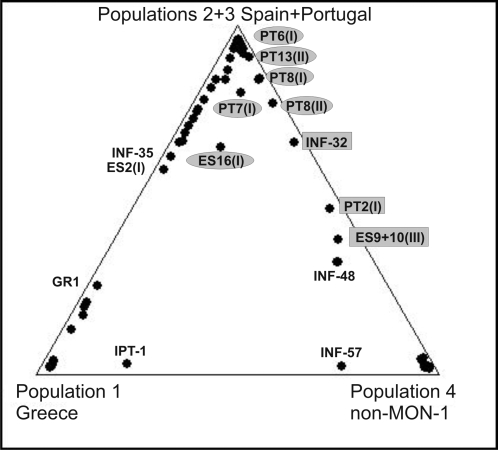
Traces of gene flow between the *L. infantum* populations shown by a triangle plot. The triangle plot of proportion of ancestry from three sources designated population 1 (Greece), population 4 (non-MON-1) and combined populations 2 and 3 (Majorca/Ibiza and mainland Spain and Portugal) as inferred from *K* = 4 in STRUCTURE analysis shows traces of gene flow between the populations. Each data point corresponds to a single strain whose proportion of ancestry from each of the three sourcesis represented by its proximity to the corresponding corner of the triangle. Strains of mixed MON-1/non-MON-1 ancestry and mosaic genotypes are labeled in grey (rectangles-most of the loci are heterozygous; ellipses-few loci are heterozygous). ES16(I) is homozygous for all loci, however representing a combination of alleles typical for different populations.

Traces of gene flow were detected between the four populations using the Bayesian algorythm (**Figure 1A** **,** **3**). Several strains could not be assigned to only one population by STRUCTURE as they showed mixed ancestry. Some strains were considered more likely to belong to one of the populations, but others had clear shared memberships for two populations (e.g. ES9(III), ES10(III), PT2(I), and INF-32) (**Figure 3** **;** **Table 3** **,** **4**). ES9(III) and ES10(III) (human isolate with its relapse) had heterozygous combinations of alleles characteristic for non-MON-1 strains and MON-1 strains (population 2) for 10 of the 14 markers. The sand fly isolate PT2(I) had such MON-1 (population 3)/non-MON-1 allele combinations in 9 markers. The remaining 5 homozygous markers were in general not discriminating between MON-1 and non-MON-1 strains. INF-32 presented also a mixture of patterns typical for non-MON-1 and MON-1 (population 3), being heterozygous in 7 markers. In addition there were several strains which also have alleles typical for both, the MON-1 and non-MON-1 group, however with MON-1 alleles clearly predominating, as the canine strain PT13(II), the sandfly strains PT7(I) and the human strains PT8(I) and PT8(II) (**Tables 3** **,** **4**). The two sandfly isolates PT7(I) and PT6(I) as well as PT8(I) and PT8(II) from Alto Douro showed an unique allele for marker Li 22–35 (118 bp) that differed significantly from the usual MON-1 alleles and rather resembled the range of non-MON-1 alleles (**Tables 4** **,** **5**). ES16(I) is homozygous for all loci, however represents a combination of alleles typical for different populations, with dominating population 3 membership (**Figure 3** **,** **Table 3**). All those strains (PT13(II), PT8(I), PT8(II), PT7(I), ES16(I)) seem to exhibit mosaic genotypes. Three of the strains showing heterozygous MON-1 and non-MON-1 alleles (PT2(I), ES9(III), ES10(III)) had in the NJ tree an intermediate position between the two respective clusters. Strain INF-32, although being heterozygous for some microsatellite markers was here part of the MON-1 cluster, suggesting the predominance of MON-1 traits.

**Table 3:** Population membership coefficients of strains of combined MON-1/non-MON-1 genotypes or other mixed ancestries

	Population 1^a^	Population 2^a^	Population 3^a^	Population 4^a^
	Greece	Ibiza+Majorca	PT+ES mainland	non-MON1
INF-32	0.013	0.030	0.653	0.303
ES9(III)	0.082	0.288	0.037	0.593
ES10(III)	0.081	0.292	0.033	0.595
PT2(I)	0.021	0.006	0.478	0.495
ES16(I)	0.215	0.065	0.602	0.117
PT8(II)	0.011	0.244	0.558	0.187
PT8(I)	0.007	0.074	0.802	0.117
PT7(I)	0.077	0.809	0.025	0.089
PT13(II)	0.004	0.929	0.013	0.054

a Populations 1–4 as inferred for *K* = 4 with STRUCTURE. PT-Portugal, ES-Spain.

**Table 4:** MLST-profiles of strains of mixed ancestry (heterozygous and mosaic genotypes) and alleles unique to Alto Douro

strain	region	MON	Lm2TG	TubCA	Lm4TA	Li 41–56	Li 46–67	Li 22–35	Li 23–41	Li 45–24	Li 71–33	Li 71–5/2	Li 71–7	CS20	LIST7031	LIST7039
			[bp]	[bp]	[bp]	[bp]	[bp]	[bp]	[bp]	[bp]	[bp]	[bp]	[bp]	[bp]	[bp]	[bp]
INF-32	Catalonia	77	138/*144*	80/**86**	*79*	*90*/**92**	80	*94*/**114**	85	*105*/**113**	105	110	100	83/85	111	207/**215**
ES9(III)	Majorca	24	142/**150**	80	75/77	*90*/**92**	**78**/80	*90*/**106**	85	*103*/109	105	**106**/110	98/100	83/**89**	111	207/**215**
ES10(III)	Majorca	24	142/**150**	80	75/77	*90*/**92**	**78**/80	*90*/**106**	85	*10*3/109	105	**106**/110	98/100	83/**89**	111	207/**215**
PT2(I)^a^	Algarve	1	142/**148**	80	*79*	*90*/**94**	80/82	100/**124**	85	*105*/**113**	105/**111**	108	**90**/100	83/**87**	111	207/**213**
ES16(I)	Majorca	1	142	80	75	*90*	80	*94*	85	*103*	105	108	98	83	111	207
PT8(II)	Alto Douro	1	**116**/140	80	75	*90*	80	*94*/118^b^	83	*105*	105	110	100	83	109/111	207
PT8 (I)	Alto Douro	1	*126*	80	*81*	*90*/**94**	80	*94*	85	119^b^	105	110	100	83	109	207
PT7(I) a	Alto Douro	1	138	80	75	*90*	**74**/80	118 b	67 b/85	*105*	105	*112*	100	83	109/111	207/**211**
PT13(II)	Alto Douro	1	138	80	nd	*90*	**74**/80	118 b	67^b^/85	*105*	105	110	100	83	111	207/**211**
PT6(I) a	Alto Douro	1	138	80	75	*90*	80	118 b	85	*105*	105	110	100	83	109	207

a *Phlebotomus* isolates;

b Alleles unique for strains from Alto Douro; bold: alleles found exclusively in non-MON-1 strains, italics: alleles found exclusively in MON-1 strains Several alleles are found in both groups-MON-1 and non-MON-1, sometimes with similar frequencies, in other cases the frequency is different in MON-1 and non-MON-1, up to the case that some alleles are dominating in one of those groups-see Table 5. nd-not defined.

**Table 5:** Allele-frequencies of each of the 14 microsatellites studied for 113 strains of *L. infantum* MON-1 and 26 strains of non-MON-1

Locus^a^	Lm2TG		TubCA		Lm4TA		Li 41-56		Li 46-67		Li 22-35		Li 23-41		Li 45-24		Li 71-33		Li 71-5/2		Li 71-7		CS20		LIST7031		LIST7039	
	Allele	F_A_	Allele	F_A_	Allele	F_A_	Allele	F_A_	Allele	F_A_	Allele	F_A_	Allele	F_A_	Allele	F_A_	Allele	F_A_	Allele	F_A_	Allele	F_A_	Allele	F_A_	Allele	F_A_	Allele	F_A_
	[bp]		[bp]		[bp]		[bp]		[bp]		[bp]		[bp]		[bp]		[bp]		[bp]		[bp]		[bp]		[bp]		[bp]	
non-MON-1^b^	116	5.8	**80**	**67.3**	**73**	**13.5**	88	3.8	**74**	**38.5**	80	1.9	**83**	**40.4**	89	3.8	**105**	**67.3**	**106**	**84.6**	90	1.9	**83**	**21.1**	**109**	**38.5**	**207**	**44.2**
	**122**	**32.7**	82	3.8	**75**	**65.4**	**92**	**11.6**	**76**	**11.5**	92	5.8	**85**	**13.4**	107	3.8	107	1.9	108	3.8	92	3.9	**85**	**15.4**	**111**	**57.7**	209	3.9
	124	5.8	**84**	**13.5**	**77**	**17.3**	**94**	**80.8**	**78**	**34.6**	100	1.9	87	5.8	**109**	**55.9**	109	7.7	**110**	**11.6**	**96**	**11.5**	**87**	**40.4**	113	3.8	**211**	**19.2**
	**134**	**11.5**	**86**	**11.6**	83	3.8	96	3.8	**80**	**15.4**	102	5.8	89	5.8	111	3.8	111	9.6			**98**	**67.3**	**89**	**17.3**			**213**	**11.6**
	136	5.8	94	3.8							**104**	**13.5**	**91**	**17.3**	**113**	**17.3**	113	7.7			**100**	**15.4**	91	5.8			**215**	**19.2**
	138	9.6									**106**	**11.5**	93	3.8	**115**	**15.4**	133	3.9									217	1.9
	140	1.9									108	7.7	95	7.7			137	1.9										
	142	1.9									112	3.8	99	5.8														
	146	5.8									114	3.8																
	148	3.8									118	1.9																
	**150**	**15.4**									120	5.8																
											**122**	**28.9**																
											124	7.7																
MON-1 total^b^	116	0.4	**80**	**99.1**	63	0.9	88	8.0	74	0.9	88	0.9	67	0.9	101	0.9	**105**	**97.3**	108	7.1	98	7.5	81	0.9	109	7.1	205	8.0
	122	0.9	88	0.9	73	0.9	**90**	**91.6**	**80**	**99.1**	**90**	**16.4**	81	0.4	103	6.2	107	2.7	**110**	**82.8**	**100**	**92.0**	**83**	**79.5**	**111**	**79.0**	**207**	**91.1**
	126	2.2			**75**	**17.9**	94	0.4			**92**	**24.3**	**83**	**12.0**	**105**	**67.7**			112	9.1	102	0.5	**85**	**19.6**	**113**	**13.9**	211	0.9
	136	0.9			**77**	**15.2**					**94**	**47.4**	**85**	**83.2**	**107**	**17.2**			124	1.0								
	**138**	**24.5**			**79**	**38.8**					96	0.4	87	3.5	109	7.1												
	**140**	**10.3**			**81**	**15.6**					98	4.0			119	0.9												
	**142**	**29.5**			83	6.7					100	1.3																
	**144**	**29.5**			85	0.9					102	0.9																
	146	1.8			87	3.1					104	1.3																
MON-1 Population^b^																												
Greece,	122	4.8	**80**	**95.5**	63	4.6	**88**	**40.9**	**80**	**100.0**	**92**	**65.9**	81	2.3	101	4.6	**105**	**100.0**	108	11.1	98	20.5	83	9.5	109	14.3	**207**	**100.0**
Turkey,	136	4.8	88	4.5	**77**	**50.0**	**90**	**59.1**			**98**	**20.5**	**83**	**25.0**	**103**	**22.7**			**110**	**27.8**	**100**	**79.5**	**85**	**90.5**	**111**	**85.7**		
Tunisia,	138	4.8			79	9.1					100	6.8	**85**	**63.6**	**107**	**72.7**			**112**	**50.0**								
Israel	140	9.5			81	13.6					104	6.8	87	9.1					124	11.1								
	**142**	**21.4**			83	18.2																						
	**144**	**54.7**			85	4.5																						
Islands Spain	**138**	**79.7**	**80**	**100.0**	**75**	**48.5**	**90**	**100.0**	74	3.1	88	3.1	67	3.1	**105**	**67.2**	**105**	**97.0**	**110**	**96.9**	98	3.1	**83**	**100.0**	109	4.7	**207**	**96.9**
Majorca+Ibiza	140	3.1			77	6.4			**80**	**96.9**	**90**	**53.1**	83	3.1	107	7.8	107	3.0	112	3.1	**100**	**95.3**			**111**	**46.9**	211	3.1
	142	17.2			79	3.2					**92**	**28.2**	**85**	**90.7**	**109**	**25.0**					102	1.6			**113**	**48.4**		
					**81**	**25.8**					94	3.1	87	3.1														
					83	4.8					102	3.1																
					87	11.3					118	9.4																
Portugal+	116	0.9	**80**	**100.0**	73	1.7	**90**	**99.1**	**80**	**100.0**	90	2.5	83	11.9	103	3.4	**105**	**97.0**	108	10.3	98	5.1	81	1.7	109	5.9	205	15.2
Spain	126	4.2			75	8.5	94	0.9			92	6.7	**85**	**86.4**	**105**	**93.2**	107	3.0	**110**	**83.6**	**100**	**94.9**	**83**	**93.2**	**111**	**94.1**	**207**	**84.8**
mainland	138	1.7			77	6.8					**94**	**89.0**	87	1.7	107	1.7			112	6.1			85	5.1				
	140	14.4			**79**	**68.6**					96	0.9			119	1.7												
	**142**	**39.0**			81	11.0					118	0.9																
	**144**	**36.4**			83	3.4																						
	146	3.4																										

a Prominent alleles (>10%) are marked as bold numbers.

b Two strains of mixed MON-1/non-MON-1 alleles have been excluded (INF-32 and PT2(I)). For ES9(III) and ES10(III) only the non-MON-1 genotype has been considered, as these strains have been identified as MON-24 by MLEE.

**Figure 4:**
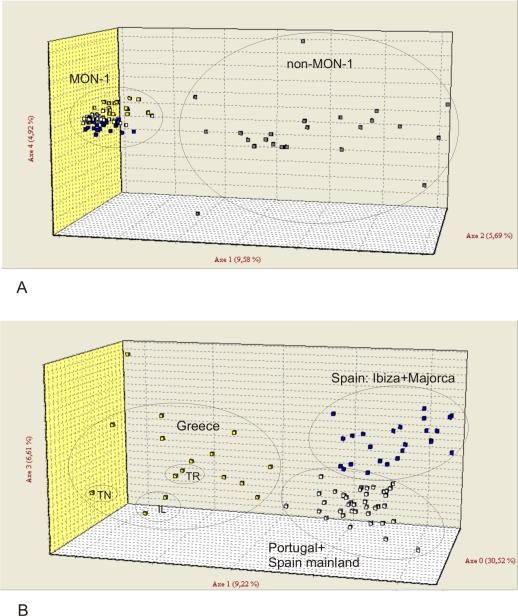
Factorial correspondence analysis (FCA) of *L. infantum* strains from the Mediterranean region. (A) All MON-1 and non-MON-1 strains included (141 strains). (B) Analysis of the three MON-1 populations (113 strains). TR–Turkey, TN–Tunisia, IL–Israel; Population 1 (Greece)–yellow squares, population 2 (Majorca+Ibiza)–blue squares, population 3 (mainland Spain+Portugal)–white squares, population 4 (non-MON-1)–grey squares.

The four populations recognized by model-based and distance-based analyses were also supported by F statistics. All *F*_ST_ values (**Table 6**) were >0.25 and significant (p = 0.0001) indicating strong genetic differentiation between the four populations. Populations 2 (Spain Majorca and Ibiza) and 3 (Portugal and Spain mainland) were the most closely related samples, an observation that is highly congruent with their geographic distribution and the maritime transport. Moreover, migration rates (*Nm*) estimates between all four *L. infantum* populations were low (**Table 6**). The graphical representation of factorial correspondence analysis (FCA) of the MLMT data (**Figure 4**) clearly mirrors the population structure parameters. The split between MON-1 and non-MON-1 strains (*K* = 2) and the higher genotypic diversity within non-MON-1 strains is apparent from **Figure 4A**. The three main MON-1 populations are clearly separated, when only MON-1 strains were analyzed (**Figure 4B**).

**Table 6:** *F*_ST_ and migration rate values (Nm) values (upper and lower triangle, respectively) for the *L. infantum* populations

Population^a^	GR+TR+IL+TN	ES Majorca+Ibiza	PT+ES mainland	non-MON-1
Greece+Turkey+Israel+Tunisia	0	0.431^b^	0.454 b	0.342 b
Spain Majorca+Ibiza	0.330	0	0.339 b	0.430 b
Portugal+Spain mainland	0.300	0.487	0	0.516 b
non-MON-1	0.481	0.331	0.234	0

a Populations as assumed by STRUCTURE *K* = 4 for 139 strains of *L. infantum* from the Mediterranean region based on 14 microsatellite markers. Strains with mixed MON-1/non-MON-1 genotype (INF-32 and PT2(I)) have been excluded. In the case of ES9(III) and ES10(III) only the non-MON-1 genotype has been considered, as these strains have been identified as MON-24 by MLEE.

b p = 0.0001 for all *F*_ST_ values. ES-Spain, PT-Portugal, GR-Greece, TR-Turkey, IL-Israel, TN-Tunisia.

### Characterization of populations: genetic diversity and differentiation, population-specific alleles

The mean number of alleles (MNA) ranged from 2.79–3.07 among the three MON-1 populations, and was 6.29 for the non-MON-1 population (**Table 7**). The proportion of polymorphic loci was 0.786 for all three MON-1 populations and 1.0 for the non-MON-1 population. The values of *H*_e_ differed between MON-1 (0.197–0.348) and non-MON-1 (0.621). The highest degree of heterozygosity was observed for the non-MON-1 population, the lowest in population 3 (MON-1 Portugal+Spain mainland). There were no indications for aneuploidy in the tested strains, since we never observed three or four peaks in the electrophoregrams, suggestive of tetra- or triploidy of the tested markers.

**Table 7:** Characterization of the 4 populations found by STRUCTURE (*K* = 4) analysis of 139 strains of Mediterranean *L. infantum*

Population^a^	Country	Region	N	P	MNA	*H*_e_	*H*_o_	*F*_IS_	NAu	I_A_
1	Greece (15/16)	Athens (7/8)	22	0.786	2.86	0.348	0.0243	0.933	7	1.291
MON-1		Crete (8/8)								(p<0.001)
	Turkey (2/2)	unknown								
	Israel (2/2)	unknown								
	Tunisia (1/1)	unknown								
	France (1/4)	Côte d'Azur (1/1)								
	Spain (1/47)^b^	Madrid (1/17)^b^								
2	Spain (28/47)^b^	Ibiza (15/15)	32	0.786	2.79	0.230	0.054	0.770	4	0.465
MON-1		Majorca (9/12)								(p = 0.001)
		Madrid (4/17)^b^								
	Portugal (3/41)	Alto Douro (3/10)								
	France (1/4)	Provence (1/1)								
3	Spain (18/47)^b^	Madrid (12/17)^b^	59	0.786	3.07	0.197	0.018	0.908	5	0.374
MON-1		Catalonia (3/3)								(p = 0.004)
		Majorca (3/12)								
	Portugal (38/41)	Lisbon (25/25)								
		Alentejo (4/4)								
		Algarve (2/3) (Nr.3 = PT2(I))								
		Alto Douro (7/10)								
	France (2/4)	Cérvennes (2/2)								
	Greece (1/16)	Athens (1/8)								
4	Spain (18)	Madrid (12)	26	1.000	6.29	0.621	0.173	0.725	46	2.522
non-MON-1		Majorca (2)								(p<0.001)
		Andalucia (2)								
		Valencia (1)								
		unknown (1)								
	Portugal (2)	Lisbon (1)								
		Alentejo (1)								
	France (3)	Pyrénées-Orientales (2)								
		unknown (1)								
	Italy (2)	Sicily (1)								
		unknown (1)								
	Malta (1)	unknown (1)								

a The two strains with mixed MON-1/non-MON-1 alleles (INF-32, PT2(I)) have been excluded. INF-35 (MON-108) is part of the MON-1 populations. In case of ES9(III) and ES10(III) only the non-MON-1 genotype has been considered, as these strains have been identified as MON-24 by MLEE.

b 3 strains from Madrid (ES1(I), ES3(I), ES6(I)) originally identified as MON-1 clearly group with the non-MON-1 population and have been considered as part of the non-MON-1 population 4. The first number in the bracket indicates the number of strains from a given focus belonging to the respective population, the second one the overall number of strains from a given focus. N, number of strains; P, proportion of polymorphic loci; MNA, mean number of alleles; *H*_o_, observed heterozygosity; *H*_e_, expected heterozygosity; *F*_IS_, inbreeding coefficient; NA_u_, number of unique alleles; I_A_-Index of association.

The inbreeding coefficient *F*_IS_ ranged from 0.770 to 0.933 among the MON-1 populations and was lowest for the non-MON-1 population (0.725). This suggests a high degree of inbreeding in all four populations, but especially in the MON-1 populations 1 (Greece, Turkey, Israel, Tunisia) and 3 (Portugal+Spain mainland). Multilocus linkage associations were highly significant for all four populations pointing to a predominantly clonal reproduction within these populations.

Allele frequencies were different in the different populations and group-specific alleles could be recognized (**Table 5**), which were congruent with the population splits. The number of group specific alleles was significantly higher among non-MON-1 strains (46) than in the three MON-1 populations (4–7). Within MON-1, population 1 (Greece, Turkey, Israel, Tunisia) had the highest number of specific alleles and it appeared as most diverse population, whereas population 3 was the least diverse.

### Geographical differentiation within MON-1 populations

The distribution of the Mediterranean MON-1 strains belonging to populations 1–3 in the respective countries and their studied endemic foci as well as the proportion of each population sampled in the respective geographical region are illustrated in **Figure 1B**.

#### Population 1: Greece (Turkey, Israel, Tunisia)

All but one strain from Greece are members of population 1, which falls into two main subclusters: one with most of the Greek strains (Greek cluster) and the other with strains from Tunisia, Israel and Turkey (East Mediterranean cluster). MLMT genotyping did not discriminate between the regions of Athens and Crete, nor between zymodemes MON-98 and MON-1. In fact, six strains originating from Athens and from Crete, and also representing the two zymodemes MON-1 and MON-98 had an identical genotype. Among these identical strains there are human as well as canine isolates.

#### Population 2: Spanish islands-Majorca and Ibiza

This population includes all strains from Ibiza, nine of the twelve strains from Majorca, four of the 17 strains from Madrid, one of the four MON-1 (including MON-108) strains from France (Provence), and unexpectedly three of the ten strains from Alto Douro, Portugal. Eight of the 15 strains from Ibiza form one subcluster in the NJ tree. The three strains from Alto Douro are closely related to each other, and, as well as the strain from France, integrated in the main population 2. There are two genotypes, identical for three strains respectively, and three genotypes shared by two strains each.

#### Population 3: mainland Spain and Portugal

This population includes all but three strains from Portugal, 18 of the 47 strains from Spain, two of the four strains from France and a single strain from Greece (GR17). All four endemic regions from Portugal are represented in this population, without any geographical correlation. Strains from Alto Douro were the most diverse. Unique alleles were found in this region in comparison with all other *L. infantum* strains for the 4 markers Lm2TG (126 bp), Li 23–41 (67 bp), Li 22–35 (118 bp) and Li 45–24 (119 bp) (**Table 4**). In addition, four loci also had unique alleles in relation to all other Portuguese strains: LIST7031 (109 bp), Lm4TA (75 bp), LIST7039 (211 bp) and Lm2TG (116, 126, 138 bp). They also show the highest degree of heterozygosity among the Portuguese strains. Nine strains from Portugal, but from different regions and host origins, had identical genotypes. Other strains, but in smaller numbers, also had indistinguishable genotypes.

### Correlation of MLMT genotypes with pathology and host specificity

No correlation was found between MLMT genotypes and host specificity or clinical manifestation (**Figure 1A** **,** **2**). Human and canine isolates were present in all MON-1 populations. Strains from *Leishmania*/HIV+ co-infected patients were represented in all four populations, whereas those few from CL cases were found only in populations 3 (mainland Spain+Portugal) and 4 (non-MON-1). The three strains isolated from Portuguese *Phlebotomus* had quite peculiar genotypes. Strain PT2(I) from Algarve had alleles characteristic for both, MON-1 and non-MON-1, as described above. The two sand fly strains from Alto Douro, PT6(I) and PT7(I), had a unique Li 22–35 allele (118 bp), found only in strains from this endemic focus and differing significantly from the common MON-1 alleles (92 and 94 bp), which resembles rather the range of non-MON-1 alleles. PT7(I) also presented both MON-1 and non-MON-1 alleles for some of the markers, albeit with MON-1 genotype dominating.

Of the 12 relapse cases, 6 occurred in MON-1, one in MON-1/MON-98, two in MON-24, another two in MON-34 and one in MON-27. Ten pairs of isolates presented identical MLMT profiles. Identical genotypes were found for a case from Greece, where the original strain was identified as MON-98 and the relapse as MON-1 [62]. In the case with four episodes, strains from episodes 1 and 2 (ES11(I)+ES12(I)) were identical but distinct from strains from episodes 3 and 4 (ES13(III)+ES14(III)), which were identical. The two pairs differed only in Lm2TG with an additional allele present in episodes 1 and 2. The case with three episodes (ES15, 16, 17(III)) had differences in two markers. The first episode isolate showed two alleles for Li 23–41 and LIST7031, the second only in Li 23–41 and the third was homozygous for both markers.

## Discussion

### Population structure and sub-structure of European *L. infantum*

The population structure of European *L. infantum* had, so far, been poorly understood, because most strains belong to a single zymodeme, MON-1. The recent development of microsatellite markers, which discriminate within this zymodeme [46], enabled for the first time to reliably address key epidemiological questions such as i) the existence of geographical subpopulations and the extent of gene flow between them, ii) the impact of zoonotic and anthroponotic transmission cycles, iii) the role of reservoir hosts and vectors in sustaining genetic diversity, iv) comparison of genetic diversity in immuno-competent and immuno-compromised hosts, v) differential identification of re-infection or relapse in treated patients, especially the immuno-compromised, vi) the role of mutation and recombination in creating genetic diversity.

We detected considerable genetic structure within European strains of *L. infantum* using model and distance-based analysis methods. The main split between MON-1 and non-MON-1 strains observed in previous MLMT studies of however only few strains of *L. infantum* [41],[46] has been confirmed. Both studies had suggested that both MON-1 and non-MON-1 strains each form a monophyletic group, and are independent as are the other populations of the *L. donovani* complex identified for *L. donovani* strains from distinct geographical regions [41]. Monophyly of the MON-1 group was also supported by recent studies based on RFLP analysis of intergenic regions of *cpb* and *gp63* [36], MLST [63],[64] and by a multifactorial genetic analysis of RFLP, MLMT, MLST and sequence analysis of non-coding regions [65], which however, all included only few MON-1 strains.

The non-MON-1 population was more diverse, with more alleles, longer branches in the tree and a broader distribution of the strains in FCA. This group may include several subpopulations, perhaps consistent with different zymodeme groups and/or geography. The position of two strains–INF-48 (Buck, Malta) and INF-57 (ISS800, Italy, Sicily) was odd and could not be resolved with confidence. In most studies using different genetic markers they showed the most distant position among the whole *L. infantum* cluster, at the basis of the non-MON-1 cluster right after the split from the *L. donovani* strains [36],[41],[63],[65]. A recent MLST study [64] placed INF-48 and INF-57 close to the MON-1 cluster. According to STRUCTURE in the present study, which does not include *L. donovani*, both strains are members of the non-MON-1 group, INF-48 had, however, partial membership in MON-1. In the distance tree INF-48 had a basal position within the MON-1 cluster, and INF-57 was a member of the non-MON-1 cluster. The phylogenetic relationships of these strains and the structure of the non-MON-1 group should be tested using a much larger set of strains including as many zymodemes as possible.

The MON-1 cluster also included other zymodemes, which must be closely related: MON-108, MON-77 and MON-98. Indeed, these and MON-1, together with four other zymodemes (MON-253, 27, 105, 72; not studied here), formed a distinct subcluster in MLEE trees, with MON-1 as the putative original zymodeme and the other zymodemes differing in the mobility of only one isoenzyme (Malic enzyme-ME, glucose-6-phosphate dehydrogenase–G6PD, NADH diaphorese-DIA, purine nucleoside phosphorylase 1-NP1, phosphoglucomutase-PGM, respectively) [22]. Different enzymatic profiles might result from post-translational modifications, as sequencing of ME which is discriminating MON-1 and MON-98 did not reveal any nucleotide differences between these zymodemes [63]. All other 20 zymodemes, 11 of which were studied here and assigned to the non-MON-1 population by MLMT, differed in 2 to 4 enzymes. Interestingly, all zymodymes of the non-MON-1 population share the 104 phenotype for MDH (Malate dehydrogenase), in contrast to zymodemes MON-1, MON-98, MON-77 and MON-108 forming our MON-1 population which share the 100 phenotype for MDH. Similarly, for NP1, all members of the non-MON-1 population present the 130 or 140 phenotype, whereas strains of the MON-1 population all present phenotype 100. Furthermore, all zymodemes belonging to the MON-1 population were also found in dogs, which is in favour of the proximity of these zymodemes. Only few zymodemes of the non-MON-1 population, MON-199, MON-34, and MON-11, have been occasionally isolated from canines.

MLMT showed that MON-1 strains are not genetically identical as previously suggested by MLEE and many different genetic markers, but represent rather families of related clones. The MON-1 group is indeed a more homogenous population than the non-MON-1 group, and characterized by a low level of heterozygosity. One could speculate that the predominance of MON-1 and the lack of diversity are due to a quite recent evolutionary history like a bottleneck followed by a rapid epidemic spread. However, its wider spread when compared to other zymodemes might be due to a better fitness and success in infecting the canine host.

Using MLMT, we identified for the first time, geographic substructures within MON-1 strains. Three clusters emerged from this study: (i) Greece/Turkey/Israel/Tunisia, (ii) Spanish Baleares islands and (iii) mainland Portugal and Spain. This observation has important epidemiological implications, as it allows the estimation of migration rates and provides a lacking biogeographical perspective for control strategies.

Microsatellite markers allowed even differentiation of MON-1 strains between endemic foci within the same country. In Spain, the majority of the strains from the mainland were genetically separated from those isolated on the two Mediterranean islands, which is most probably related to the existing geographical barrier. Some strains from the mainland presented a genotype typical for the islands and *vice versa*, which can be explained by the transfer of parasites due to travel of infected persons or dogs. Analysing RFLP of kDNA [37] concluded that *L. infantum* from Majorca constituted a clonal population, though no mainland strains were included in that study. Whether strains from Catalonia (the three analysed herein grouped together in all distance trees) differ from other mainland strains should be re-investigated with larger sample sizes.

In Portugal, no subclusters correlating with the four regions were identified, which is congruent with a recent RFLP study on kDNA [38]. These authors explain the lack of focus-specific genotypes by the small size of the country and the frequent migration between foci. According to our data, Alto Douro seems to be the most divergent endemic focus in Portugal, followed by the Algarve. Seven strains from Alto Douro, three human (PT8(I), PT8(II), PT10(I)), two canine (PT13(II), PT14(II)) and two *Phlebotomus ariasi* isolates (PT6(I) and PT7(I)), presented unique alleles for 8 of the 14 markers used, in some cases identical to those found only in non-MON-1 strains, in other cases resembling the repeat range typical for non-MON-1 strains and in three cases alleles were found exclusively in Alto Douro. The human isolate PT8(II) had heterozygous loci combining the specific Alto Douro alleles and the alleles typical for all other strains of the mainland ES/PT population. Some of the strains from this focus (PT8(I), PT7(I), PT8(II), PT13(II)) showed for single loci alleles typical for non-MON-1.

The Greek MON-1 strains could not be further differentiated regardless of whether they came from the Athens area or the island of Crete. The strains from Turkey, Israel and the single strain from North Africa (Tunisia) grouped with those from Greece but seem to represent a distinct genotype. This needs, however to be confirmed on a larger sample set from those regions.

### Correlation with hosts and clinical manifestation

Two important interrelated epidemiological questions are the impact of zoonotic and anthroponotic transmission cycles and the role of reservoir hosts and vectors in sustaining genetic diversity. In our study no general relationships between MLMT genotypes, host and reservoir were detected. Human and canine isolates were present in all of the three main MON-1 populations. Moreover, in all MON-1 groups cases of identical MLMT profiles were identified for canine and human (immunocompetent and immunocompromised) isolates, thus pointing to transmission of the same parasite between humans and dogs, the domestic host. Such groups of identical genotypes were found in each of three MON-1 populations. The single isolate from a fox, representing a sylvatic host, did not show any special position within the MON-1 strains. An interesting question is why only a single predominating zymodeme is found in dogs–MON-1. We could, however observe a considerable amount of diversity among the canine MON-1 isolates, even in such a small territory as Ibiza, where most of them had different genotypes. A complete transmission cycle and the vector status of *P. ariasi* was confirmed by detecting nearly identical genotypes for human, canine and sand fly isolates from the Alto Douro focus. Such complete transmission cycles were also shown recently using kDNA PCR-RFLP [37],[38].

Importantly, our data did not advocate for a correlation between MLMT profile and clinical manifestation. VL was present in all populations, whereas CL only in populations 3 (mainland Spain+Portugal) and 4 (non-MON-1). VL/HIV+ co-infections were represented in all four populations and no special cluster of VL/HIV+ strains was observed. This is in contrast to reports from Spain [37],[66] and Portugal [38], which found kDNA PCR-RFLP patterns, which were associated with the immune status (HIV+ immuno-compromised and immuno-competent) of the patients. This has been attributed to the existence of anthroponotic transmission cycles due to needle sharing among drug users. Whether these contradictory results are caused by the use of different DNA types, nuclear and kinetoplast minicircle DNA, remains to be elucidated. By increasing *K* in STRUCTURE analysis we observed in *K* = 5 a split of the non-MON-1 population, present also in the distance-based tree. Interestingly, all newly described zymodemes, found exclusively in HIV+/leishmaniasis co-infections [9],[30],[67] are members of the same subgroup. We also found that the majority of strains with these zymodemes were heterozygous suggesting a relationship between anthroponotic syringe transmission cycles and recombination events.

### Detection of relapses and re-infections

Identical MLMT genotypes were found for strains isolated from different episodes in 10 out of the 12 relapse cases. For the two cases presenting different MLMT profiles in the respective episodes it is possible that the first infection was due to two strains, of which one has been eliminated during the first curse of treatment. Another possibility is re-infection with a new strain. Because microsatellite markers did not detect an individual MLMT fingerprint for each strain, re-infection can only be detected if strains from successive episodes have different genotypes. Otherwise, *de-novo* infection with an identical genotype circulating in the same focus cannot be excluded. RFLP analysis of minicircle kDNA has been successfully applied for monitoring VL outbreaks among intra-venous drug users and for differentiation between relapses and re-infections [13],[14]. However, because of the multicopy nature of minicircle kDNA, the RFLP profiles are very complex, difficult to interpret and problematic for inter-laboratory comparisons. The stability of the pattern in the course of treatment is also still under question. Microsatellite marker stability during long term cultivation and animal passages has been confirmed (data not shown). Nevertheless, MLMT and kDNA PCR-RFLP are the most discriminative methods available to date for strain fingerprinting [42].

### Gene flow and recombination

In this study, some amount of gene flow was detected between and within all four populations. This became evident in the STRUCTURE plots where some strains could not be clearly assigned and had membership coefficients for more than one population and was attributed to the presence of alleles typical for more than one population either in homozygous or heterozygous combinations in single strains.

Inbreeding coefficients and indices of association as a measure of multilocus linkage disequilibrium (LD) pointed to a predominantly clonal propagation particularly of all populations found for MON-1, but also the non-MON-1 population, as previously suggested [68]–[72]. However, it does not rule out the possibility of occasional genetic recombination, as supported here by the occurrence of strains of potential mosaic and heterozygous genotypes, even between MON-1 and non-MON-1 populations. Two explanations are likely: (1) mixed infections, (2) hybrid strains/recombination. No indications for aneuploidy or polyploidy, asexual mechanisms leading to genetic polymorphism during reversion to the normal ploidy and having been described to occur in *Leishmania* [73], were obtained in our study.

By using highly discriminatory markers on 141 strains of *L. infantum*, among them 119 MON-1 strains, we tried to minimize the impact of statistical type II errors (too few strains, poorly discriminating markers) which probably have led to underestimation of recombination events in *Leishmania* in previous studies, as was suggested among others by Tibayrenc [69],[74].

Significantly, all three sand fly isolates but only five of the many human isolates and a single canine isolate were of mixed ancestry. Whilst the *Phlebotomus perniciosus* isolate PT(II)2 had for most of the markers alleles characteristic for both, MON-1 and non-MON-1, in the second sand fly isolate PT7(I), the canine strain PT13(II) and the human isolates PT8(II) and PT8(I) MON-1 type alleles dominated. Furthermore, the two sand fly isolates from Alto Douro PT7(I) and PT6(I) had a unique allele for marker Li 22–35 that differed significantly from the usual MON-1 alleles and rather resembled the range of non-MON-1 alleles.

*Leishmania* hybrids have been reported in the literature, even in cloned parasites [75]–[79]. The mechanisms underlying hybrid formation are, however still unknown. The observation of a higher number of putative recombinant genotypes in the vector could suggest that recombination occurs in the vector. Alternatively, vectors could transmit strains that are infective, but normally do not cause disease and that are, therefore, not isolated from the vertebrate host. Preliminary data on sand fly isolates indicate that vectors might play a role in sustaining genetic diversity.

Mixed infections as a basis for recombination events are also conceivable in HIV patients, especially when parasites are transmitted by needle sharing [79]. That most of the new *L. infantum* zymodemes, almost exclusively detected in HIV co-infected patients [9],[24],[30],[37],[67],[80], are heterozygous favours this hypothesis. Recently, a substantial number of heterozygous sites and mosaic genotypes were identified in strains of *L. donovani* and *L. infantum* [64]. These authors concluded that genetic exchange would be the most plausible explanation for their data and that the importance of recombination events in *Leishmania* has been underestimated so far, which is supported by the present MLMT data.

### Conclusions

In the present study, for the first time several epidemiological questions linked with the MON-1 zymodeme could be addressed using fast evolving microsatellites. These markers are hypervariable, genetically neutral and co-dominant and are therefore ideal to detect fine scale and recent genetic structure. Last but not least, the analyses could be performed directly from biological material with a high throughput. The results obtained in different laboratories are comparable and can be stored in a database. For a comprehensive understanding of *L. infantum* epidemiology strains from all endemic foci around the Mediterranean have to be studied, and a good sampling strategy has to be applied (e.g. more sand fly isolates, other viscerotropic and dermotropic zymodemes, study of foci with significant enzymatic polymorphism etc.). The basis for such a study is the present work, which can now be completed by the data of all those mentioned strains.
